# *Toxins*: State of Journal Report, 2016

**DOI:** 10.3390/toxins7124895

**Published:** 2015-12-15

**Authors:** Vernon L. Tesh, Bryan Grieg Fry

**Affiliations:** 1 Department of Microbial and Molecular Pathogenesis, Medical Research and Education Building, Room 3002, College of Medicine, Texas A & M University System Health Science Center, 8447 State Highway 47, Bryan, TX 77807, USA; tesh@medicine.tamhsc.edu; 2 Venom Evolution Laboratory, School of Biological Sciences, University of Queensland, St. Lucia, QLD 4072, Australia; bgfry@uq.edu.au

In the “Message from the Editor-in-Chief” posted on the *Toxins* website (see www.mdpi.com/journal/toxins/toxins-flyer.pdf), we wrote: “The editorial board and staff of *Toxins* are dedicated to providing a timely, peer-reviewed outlet for exciting, innovative primary research articles and concise, informative reviews from investigators in the myriad of disciplines contributing to our knowledge on toxins. We are committed to meeting the needs of the toxin research community by offering useful and timely reviews of all manuscripts submitted”. Now, on behalf of the editors-in-chief, the editorial board, and the editorial staff of *Toxins*, we are pleased to present the following information in support of meeting our charge and commitment to our colleagues.

**Figure 1:**
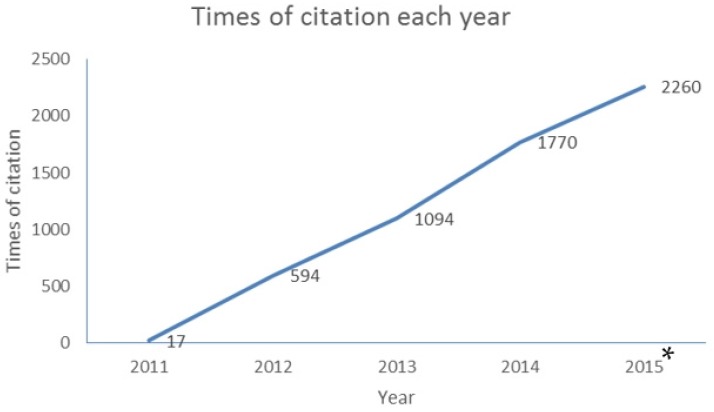
Growth in citations of *Toxins* papers since 2011. * Data collected as of November 2015 from Web of Science.

**Figure 2:**
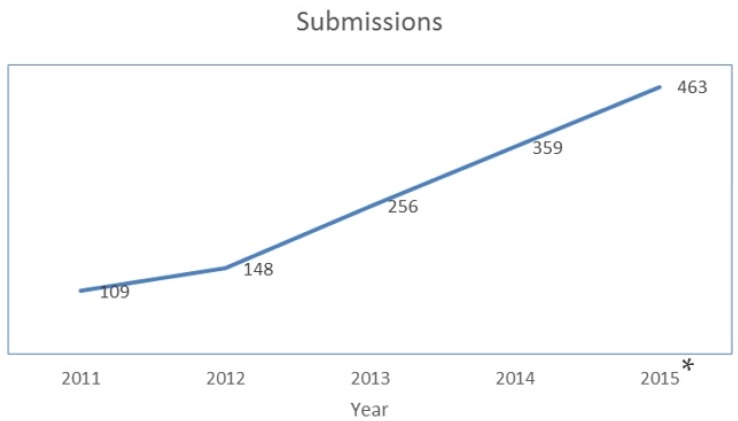
Growth in number of manuscripts submitted to *Toxins* since year 2011. * Data collected as of November 2015 from Web of Science.

**Figure 3:**
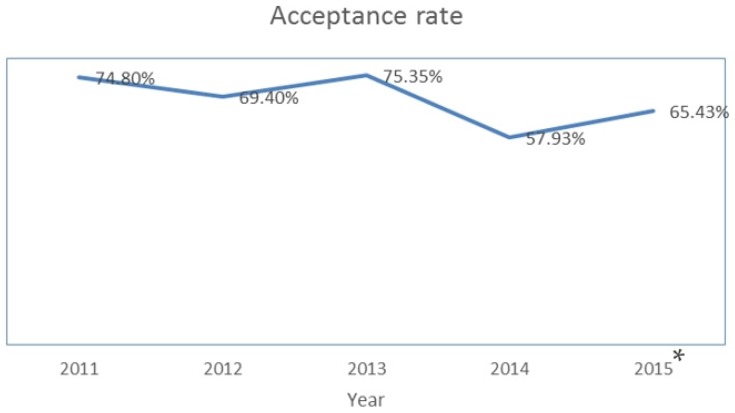
Acceptance rates for manuscripts submitted to *Toxins* since year 2011. * Data collected as of November 2015 from Web of Science.

First, it is our pleasure to announce that the journal impact factor for 2014 is 2.938, with a five-year impact factor of 3.283. These rankings place *Toxins* 28th out of 87 journals covering the fields of toxicology. The growth in the number of citations from *Toxins* is impressive (see Figure 1). As might be expected, coincident with the increase in our impact factor, the numbers of manuscripts submitted to *Toxins* has also increased, from 256 in 2013 to 359 in 2014 and 463 in 2015 (see Figure 2). Over the same period, our acceptance rate has changed from 75.35% (2013) to 57.93% (2014) and 65.43% (see Figure 3). Finally, half of the papers were published within 60 days in 2015. Taken together, these numbers reflect, first, the hard work of investigators submitting their best work to the journal; second, the hard work of our editorial board members and *ad hoc* reviewers in providing timely and insightful reviews; and third, the expert assistance of the editorial staff to produce the high quality presentation expected of papers published in a high impact journal.

In response to input from *Toxins*’ authors, reviewers and editors, we have decided to make the following changes to the editorial process. Since launching in 2009, *Toxins* has used single blind peer-review, in which authors’ names are known to reviewers but the reviewers’ identities are unknown to the authors. Effective on the first issue of 2016, a double blind peer-review procedure will be used for all the manuscripts submitted to *Toxins*: authors’ names will not be known to reviewers until the paper is published. The rationale for this change is a growing concern that bias—both deliberate and unconscious—can affect the review process. We feel that a double-blind review procedure will help preserve objectivity in the peer-review process. Technical details on preparation of a manuscript for double-blind peer-review can be found in the Instructions for Authors section of the *Toxins* website: http://www.mdpi.com/journal/toxins/instructions. In response to author and reader feedback, we have also updated the layout of published articles. PDF files remain the predominant way that research articles are read and we want to ensure that *Toxins* produces attractive and readable papers. The new layout condenses a great deal of information in the front matter and simplifies a number of elements throughout the design. We have also updated some of the technical aspects of production to give a higher quality final product.

**Figure 4:**
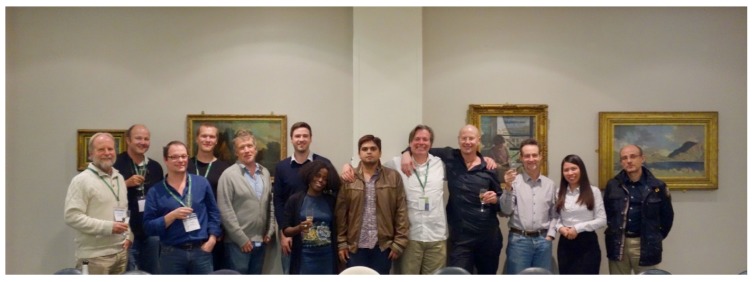
*Toxins* editorial board meeting, Oxford, England, Fall 2015.

During the course of discussions at the *Toxins* Editorial Board meeting in Oxford last fall (Figure 4), the need for a standardized nomenclature for snake venoms became clear. To assist investigators in the field, Associate Professor Bryan Grieg Fry, *Toxins* Section Editor-in-Chief for Animal Toxins, has agreed to review the nomenclature of snake venoms and recommend a standardized scheme to characterize these venoms that will greatly benefit investigators in the field.

**Figure 5:**
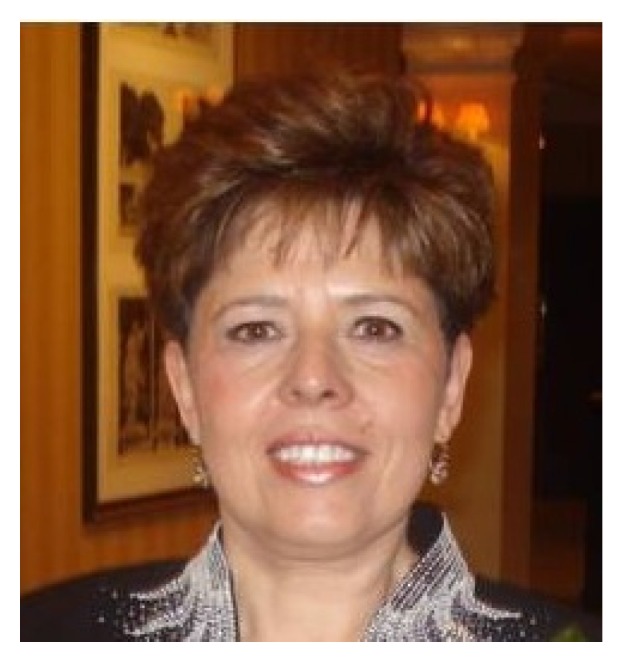
Dr. Nilgun Tumer, Section Editor-in-Chief for Plant Toxins.

It is our pleasure to welcome Dr. Nilgun Tumer (Figure 5), Professor in the Department of Plant Pathology, Biotechnology Center for Agriculture and the Environment at Rutgers University, as the new Section Editor-in-Chief for Plant Toxins. Dr. Tumer will help us cover an important research area of toxinology. We are also in the process of identifying “Leading Opinions” editors who will assist with soliciting and evaluating review articles from recognized leaders in various fields of toxinology.

MDPI has agreed to partner with Publons (https://publons.com), a company providing recognition for peer reviewers. Publons will make publicly available details of which journal scholars have reviewed for (without identifying the specific paper). The reviewer can then log onto the Publons website and decide if they wish to make more details about their review available. We believe that providing credit for reviewers will motivate them to accept review invitations, and that this is also an appropriate method for publicly acknowledging their contribution, which is almost always anonymous and difficult to quantify.

MDPI Publishing and *Toxins* are making a significant investment in the future of toxin research by supporting two Travel Awards for post-doctoral fellows or graduate students to attend a conference in 2016 to deliver an oral or poster presentation on research related to toxins. We trust you encouraged your students to participate in this competition. Look for the announcement of the winners next month.

In closing, we hope this brief update on the state of the journal demonstrates the desire of the editorial board and staff of *Toxins* to meet the needs of the toxin research community. We are always amazed by the creativity and inventiveness of our colleagues in the world of toxins, and by all means, do not hesitate to contact us, or any member of the editorial board or staff, to let us know how we can better meet your needs.

